# Evolutionary Approach to Optimal Oil Skimmer Assignment for Oil Spill Response: A Case Study

**DOI:** 10.3390/biomimetics9060330

**Published:** 2024-05-30

**Authors:** Yong-Hyuk Kim, Hye-Jin Kim, Dong-Hee Cho, Yourim Yoon

**Affiliations:** 1 School of Software, Kwangwoon University, 20 Kwangwoon-ro, Nowon-gu, Seoul 01897, Republic of Korea; yhdfly@kw.ac.kr; 2 TmaxBI, 29 Hwangsaeul-ro, 258beon-gil, Bundang-gu, Seongnam-si 13595, Gyeonggi-do, Republic of Korea; ovoa22@gmail.com; 3 Munhwa Broadcasting Corporation, 267 Seongam-ro, Mapo-gu, Seoul 03925, Republic of Korea; dh_cho@mbc.co.kr; 4 Department of Computer Engineering, Gachon University, 1342 Seongnamdaero, Sujeong-gu, Seongnam-si 13120, Gyeonggi-do, Republic of Korea

**Keywords:** oil skimmer assignment, genetic algorithm, surrogate model, resource allocation

## Abstract

We propose a genetic algorithm for optimizing oil skimmer assignments, introducing a tailored repair operation for constrained assignments. Methods essentially involve simulation-based evaluation to ensure adherence to South Korea’s regulations. Results show that the optimized assignments, compared to current ones, reduced work time on average and led to a significant reduction in total skimmer capacity. Additionally, we present a deep neural network-based surrogate model, greatly enhancing efficiency compared to simulation-based optimization. Addressing inefficiencies in mobilizing locations that store oil skimmers, further optimization aimed to minimize mobilized locations and was validated through scenario-based simulations resembling actual situations. Based on major oil spills in South Korea, this strategy significantly reduced work time and required locations. These findings demonstrate the effectiveness of the proposed genetic algorithm and mobilized location minimization strategy in enhancing oil spill response operations.

## 1. Introduction

Oil spill accidents [1] pose a grave threat to the environment, causing substantial ecological harm and economic losses that persist over extended periods. Effective planning is essential to prevent such incidents, detect them promptly, and respond swiftly when they occur. Central to an efficient response strategy is the optimal allocation of resources, which not only mitigates environmental damage but also minimizes the social and economic impacts. Previous research by Psaraftis et al. [2] underscored the importance of quantifying regions’ response capabilities to oil spills, offering a model applicable to areas with heightened spill risks, such as New England.

Given South Korea’s geographical disposition as a peninsula surrounded by sea, the nation has established control resources across 16 locations to manage oil spills effectively. Optimizing the allocation of these resources is crucial, with past studies, such as the study by Yun et al. [3], exploring manual allocation methods by domain experts. In this paper, we introduce a novel approach to optimize the assignment of oil skimmers for spill response, utilizing a simulation-based evaluation method tailored to real-world scenarios.

Our study presents two assignment strategies: one minimizing work hours based on South Korea’s regulations and another minimizing skimmer usage. To determine these assignments, we employ a genetic algorithm (GA) [4,5], which has been representative among swarm and evolutionary computation methods [6], and compare the results with current practices in South Korea through simulations. However, the complexity and time-intensiveness of simulation-based evaluations necessitate alternative approaches.

Therefore, in our second study, we propose a surrogate model that employs a deep neural network (DNN) [7,8] to expedite resource allocation optimization. Building upon prior research by Shin and Kim [9], we aim to substitute simulation-based evaluations with an efficient surrogate model, comparing its performance and optimization results to precedent studies.

Furthermore, our third study addresses the inefficiencies of current assignment methods by minimizing the number of locations mobilized in response to oil spills. Conducting experiments based on 19 oil spill scenarios in South Korea, we validate our strategies by comparing mobilized location minimization with the original strategy of minimizing work time.

By elucidating these advancements in oil spill response optimization, this study contributes to enhancing preparedness and efficacy in managing environmental disasters, particularly in maritime jurisdictions such as South Korea. Our approach is designed to be applicable beyond the specific case study presented in South Korea. While the study draws on data and examples from South Korea, the methodology and principles can be adapted and applied to similar contexts in other regions or industries.

The remainder of this paper is organized as follows. In Section 2, we introduce prior work using GAs for resource allocation problems. In Section 3, we describe oil skimmer assignment problems. We propose our methods in Section 4 and present experimental results in Section 5. Finally, we conclude the paper in Section 6.

## 2. Prior Work Related to Resource Allocation

Resource allocation optimization [10,11] is a fundamental challenge encountered across various sectors, ranging from water resources [12] to manufacturing [13] and beyond. This problem entails the efficient allocation of limited resources [14] in accordance with specific contextual requirements. However, resource allocation optimization problems typically manifest as high-dimensional combinatorial optimization challenges, rendering exhaustive exploration of the solution space impractical for finding the global optimum. Consequently, practitioners often resort to heuristic search methods [15], leveraging fitness evaluation to identify optimal solutions. Given the diverse array of situations and constraints inherent in resource allocation optimization, the utilization of metaheuristic algorithms [16] that offer flexible solution search mechanisms proves more suitable than traditional heuristic search approaches.

To address such complex real-world resource allocation problems, researchers have explored the application of prominent metaheuristic techniques, such as GAs. Dai and Wang [17] proposed an optimization model for grid service allocation problems, centered on grid service reliability evaluation. Leveraging GAs, they obtained nearly optimal solutions, particularly in scenarios devoid of correlated faults.

Similarly, Lavric et al. [18] employed GAs to optimize multi-resource systems featuring pollutants and low-level resources. Their approach factored in pollution levels alongside constraints on input and output devices, showcasing the superiority of GA-based methodologies over mathematically programmed methods for their test cases.

Furthermore, Guerrero et al. [19] proposed a GA-based approach for resource allocation of containers within cloud architecture. Their algorithm, operating across four optimization objectives that encompassed cloud clusters, containers, microservices, and provisioning, bolstered system performance and resilience. By optimizing resources genetically, they effectively addressed container allocation and elasticity challenges, showcasing superiority over existing container management policies implemented in platforms like Kubernetes.

## 3. Oil Skimmer Assignment Problems

**Figure 1:**
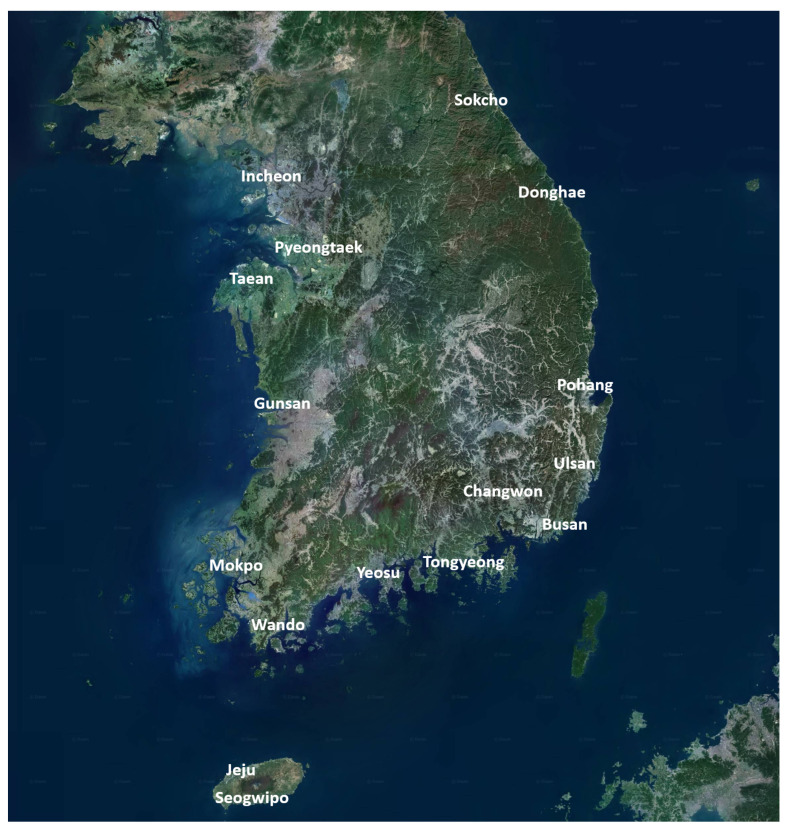
The 16 locations for storing oil skimmers in South Korea span latitudes ranging from $33^{\circ }$ N to $39^{\circ }$ N and longitudes ranging from $124^{\circ }$ E to $131^{\circ }$ E.

**Figure 2:**
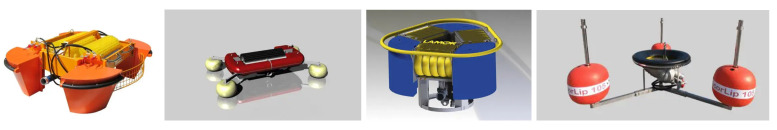
Example oil skimmers (two brush oil skimmers and two weir ones) to be assigned at each location (photos from https://www.nauticexpo.com, accessed on 27 May 2024), in order from left: SPRUT-2 (capacity 30 $m^{3}$/h), ScorSkim60 (capacity 86 $m^{3}$/h and LHW 2.3 m × 0.5 m × 1.5 m), LHS50/70 (capacity 272 $m^{3}$/h and LHW 1.6 m × 1.2 m × 1.7 m), and ScorLip135 (capacity 105 $m^{3}$/h and LH 1.9 m × 0.8 m).

We determined the maximum amount of oil that can be spilled at each location based on a technical report by Yun et al. [3], as well as the work hours that conform to South Korea’s regulations. Figure 1 shows 16 locations for storing oil skimmers in South Korea. Figure 2 shows example oil skimmers [20] to be assigned at each location. As mentioned in the introduction, we designed two strategies. The first one minimizes work hours to meet the aforementioned regulations, ensuring that a limit of 24 h is not exceeded at each location. This strategy reassigns oil skimmers currently assigned in South Korea, thereby minimizing the time required to respond to and resolve an oil spill accident. The second strategy minimizes the total capacity of the oil skimmers used in the response. We found a minimal oil skimmer capacity that facilitates GA-based optimal oil skimmer assignments for oil spill response via a binary search.

To construct experiments that incorporate both real-world constraints and region-specific optimal consumption, we based our study on the final report of the National Disaster Response Capability Assessment Study written by Yun et al. [3]. In this report, resource allocation for disaster response is optimized by dispersing oil skimmers, equipment designed to recover spilled oil, across 16 locations in South Korea to effectively respond to marine pollution incidents. The objective is to find the optimal placement of oil skimmers to facilitate offshore oil recovery using the deployed skimmers. Given that resource allocation for disaster response is a typical real-world problem, it is subject to various constraints. In this paper, unless otherwise specified, we assume the maximum possible amount of oil that can be spilled at each accident location of South Korea in the event of an oil spill scenario.

However, deriving, evaluating, and optimizing solutions for real-world problems without clear evaluation methods is exceedingly challenging. In this study, to address this issue, we devised simulations similar to actual oil spill scenarios. Simulations formulated to minimize the time required to recover the target amount of maximum spillage can be expressed as follows.

The function f(x,i) represents the time taken to recover the target amount $Q_{i}$/3, corresponding to a third of the spillover amount $Q_{i}$ at accident location *i* with the deployed skimmers $x=(x_{1},x_{2},\ldots ,x_{n})$, where *n* is the number of skimmer-deployed locations. We assume that each $x_{i}$ is a nonnegative real number. Therefore, the objective function of a GA utilizing this simulation can be expressed as follows: minimize $\sum_{i=1}^{S}f\left(x,i\right)$, where *S* is the number of accident locations (scenarios).

The hardness of the problem depends on the complexity of computing the function f(x,i). If f(x,i) is linear (as a simple case), the problem can be solved in polynomial time by a linear programming technique [21]. But, if f(x,i) is close to a black-box function (for which, to obtain the value, simulation is needed) computed in polynomial time, the problem might become NP-hard since it is intractable to consider all the feasible assignments (refer to [22,23,24]).

Now, to formulate f(x,i), we define some symbols and functions. $t_{ij}$ indicates the time taken for the vessel with oil skimmers to move from location *i* to location *j*. When an oil spill accident occurs at location *j* and oil skimmers are stored at location *i*, the recovery job can start after the time $t_{ij}$. We have a constraint to recover the target amount of the spillover within 3 days, with the daily condition of 8 hours of available work time between 8:00 a.m. and 6:00 p.m. The function h(t) represents the time not working while mobilizing the vessel, where *t* is the vessel moving time, and the start time of the recovery job is 12:00 p.m. For example, when an accident occurs at 12:00 a.m., h(9)=1. Let the function $g(t,t_{s})$ be the time not working when *t* is the vessel moving time, and $t_{s}$ is the start time of the recovery job. Then, $g\left(t,t_{s}\right)=h\left(t+t_{s}\right)-h\left(t_{s}\right)$. For example, if the vessel moving time is 3 h and the start time of the recovery job is 9:00 a.m., g(3,9)=h(3+9)−h(9)=4−1=3.

Following the report by Yun et al. [3], we used an efficiency coefficient α of 0.2 and a vessel mobilization rate β of 1/3. We assume that the recovery job starts at 8:00 a.m. and ends at 6:00 p.m., and the total daily work time of the recovery job is 10 h, but the time efficiency γ=0.8, which means that the actual daily working time becomes 8 h. Under these assumptions, f(x,i) becomes

(1) $$\begin{array}{c} \min\left\{t\;|\;\frac{Q_{i}}{3}-\alpha \beta \gamma \sum_{j=1}^{n}\max\left(t-g\left(t_{ij},T_{0}+t_{ii}\right),0\right)x_{j}\leq 0\right\}, \end{array}$$

where $T_{0}$ is the time when the accident occurred. Then, the time complexity of our simulation to compute $\sum_{i=1}^{S}f\left(x,i\right)$ is $O(STn^{2})$, where *S* is the number of accident locations (scenarios), *T* is the number of simulated work hours, and *n* is the number of skimmer-deployed locations. In our experiments, *S* is set to 1 or 16, and *T* and *n* are 24 and 16, respectively.

In the problem, to minimize the number of mobilized locations, we use the simulation function shown in Equation (2). This then solves for the recovery amount $q_{jk}$ with work time *k* for the oil skimmers at the *j*-th location, subsequently returning the minimum number of locations *l* that satisfy the conditional equation.

(2) $$\begin{array}{c} \min(l)\\ \mathrm{subject}\;\mathrm{to}\;\;\;\;\frac{Q_{i}}{3}-\alpha \beta \cdot \max\left(\sum_{j=1}^{l}\sum_{k=1}^{T}q_{jk}\right)\leq 0, \end{array}$$

where 0<l≤16 and T=24 (h).

## 4. Proposed Methods

### 4.1. Genetic Algorithm

We developed a novel GA for determining optimal oil skimmer assignments, focusing on a crucial aspect known as repair operation. The proposed GA incorporates a method for modifying chromosomes to ensure feasible solutions for oil skimmer assignment, obtained through a two-stage repair operation. In the initial stage, adjustments are made to the total capacity of the oil skimmers to meet specified constraints. If the sum of assigned oil skimmer capacities exceeds or falls short of the capacity limit, an arbitrary location is selected, and capacities are adjusted accordingly. In the subsequent stage, further adjustments are made to ensure adherence to capacity limits for specific locations, thereby preventing the allocation of all oil skimmers to locations with the highest potential spills.

In addition to the repair operation, the evaluation was structured to align with current South Korean regulations. Oil skimmers deployed to each location undergo evaluation through the simulation of an oil spill accident. The evaluation accounts for the expected maximum amount of oil spill at each location, occurring independently of those at other locations. Here, “independent accident” denotes non-simultaneous occurrence of oil spill accidents. The evaluation metric is the amount of work hours needed to recover one-third of the spilled oil from the sea using the presently assigned oil skimmers. Additionally, at each location, a certain amount of time is needed to transport the oil skimmers to the accident site via a vessel.

The operations of the GA designed in this study are as follows. When the GA is executed, it selects two different assignments from one population, creates a new assignment through crossover operation between the two assignments, and undergoes a mutation process that changes the assigned amount with a certain probability. The newly created assignment then goes through a repair process to satisfy the given constraints, receives an evaluation for fitness regarding problem-solving, and is replaced if it has a higher fitness compared to the best assignment from the previous generation. The detailed parameters of the GA are given in Table 1.

### 4.2. Surrogate Model for Evaluation

Optimizing oil skimmer assignment must take into account many real-world constraints, making it necessary to evaluate based on simulation. However, evaluating assignments through simulation is complex and time-consuming, as shown in [27]. In this subsection, we present a surrogate model that replaces the evaluation function of assignments based on simulation of oil spill accidents with DNNs and compare it with simulation over a GA. A surrogate model approximates the objective function, which has the advantage of saving time over the original objective function. When applied to simulation-based resource allocation, our surrogate model output a fitness 0.8% higher than that of the simulation in the strategy of minimizing the work time due to oil spill accidents, and the assignment result was 2.5% higher than the current assignment. Additionally, it demonstrates that the strategy for minimizing the total capacity of oil skimmers for oil spill response can be reduced by about 15% from the current assigned capacity of 225 hectotonnes (ht). Moreover, it shows that the computation time required to derive one assignment in the process of optimizing the assignment of oil skimmers for oil spill response improved by about 61%.

A surrogate model deduces by approximating the objective function, saving computation time compared to the original objective function. The methodology of our surrogate-assisted GA is as follows. Initial solutions for the oil skimmer assignment of capacity between the upper and lower limits are generated. Among the population, two parents are chosen, which then undergo crossover and mutation. As the solution that goes through crossover and mutation may not satisfy the constraints, we ensure that the constraints are satisfied through repairs. The solution generated through this procedure is evaluated by our DNN-based surrogate model and replaced. This procedure is repeated over 60,000 generations. The genetic operators used are the same as those in the GA given in Section 4.1 (see Table 1).

To construct the DNN-based surrogate model for evaluation, 10,000 assignments yielded by the GA, which were analyzed by a clustering technique (as shown in Appendix A), and 10,000 samples of randomly allocated assignments were collected. The constraints for the oil skimmer assignment were designed according to South Korea’s regulations following the study by Yun et al. [3]. For the simulation, the time of oil spill accidents was set to be 8 a.m., and the speed of the vessel for the oil skimmer supply was assumed to be 10 knots. To approximate the objective function, we recursively analyzed the data using a DNN with 100 epochs and a batch size of 5. The generated DNN model was validated by 10-fold cross-validation, with the results shown in Table 2. Additionally, the evaluation operator was constructed by calculating the fitness based on the results.

### 4.3. GA-Based Mobilized Location Minimizer

**Figure 3:**
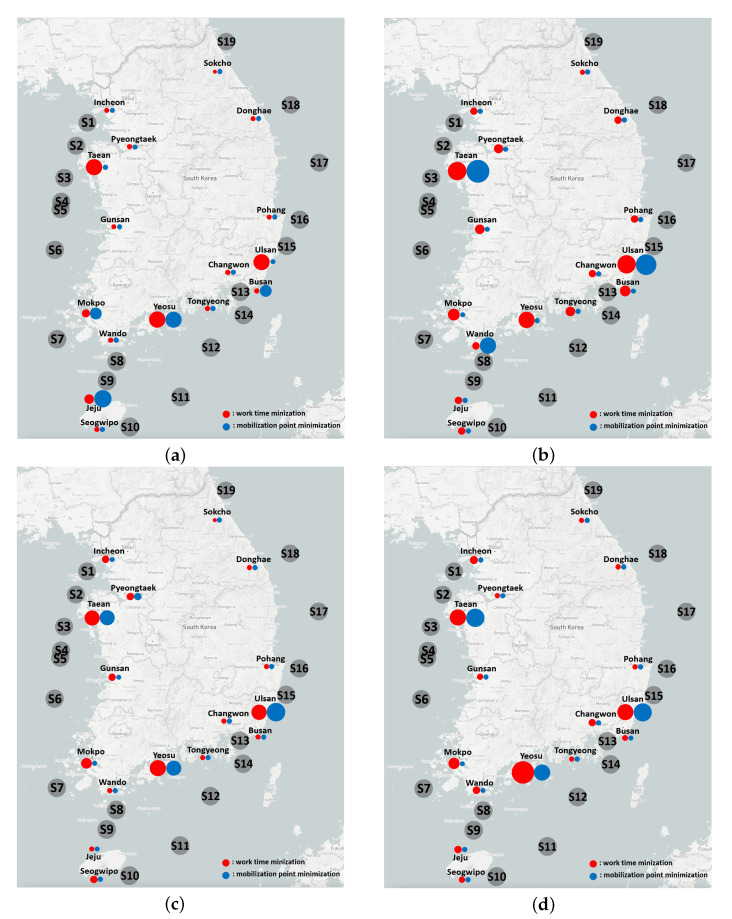
The nineteen scenarios of oil spill accidents from $S_{1}$ to $S_{19}$ and the 16 locations storing oil skimmers with the assignment plan in South Korea. (**a**) Recovery starting at 8:00 a.m. with the vessel at a speed of 10 knots; (**b**) Recovery starting at 8:00 a.m. with the vessel at a speed of 5 knots; (**c**) Recovery starting at 12:00 p.m. with the vessel at a speed of 10 knots; (**d**) Recovery starting at 12:00 p.m. with the vessel at a speed of 5 knots.

The mobilized location minimization strategy for the new oil skimmer assignment plan utilizes 19 oil spill scenarios that have not been previously used (see Appendix B for details of the scenarios). The scenario areas were selected to represent the largest spills that could occur at various locations without bias toward one area in South Korea’s sea. In Figure 3, the 19 black areas represent the locations of the accidents based on the scenarios, while the 16 red areas represent the locations of oil skimmers distributed throughout South Korea.

The objective function for validating the assignment generated by the GA is computed through simulations. These simulations evaluate the recovery up to a target amount (one-third of the spilled oil) within a given work time (24 h), while minimizing the number of locations to be mobilized. The GA required approximately 11.19 s to derive its assignment plan. The evaluation function of the GA, as shown in Equation (2), minimizes the total number of mobilized locations required for recovery in the *i*-th scenario $sce_{i}$ of *m* scenarios based on the *p*-th assignment of the *g*-th generation *c*(*g*,*p*):

(3) $$\sum_{i=1}^{m}\mathrm{simulation}\left(sce_{i},c\left(g,p\right)\right)$$

By further optimizing the total assigned capacity through the combination of the mobilized location minimization strategy with the GA, we anticipate a gradual reduction in the number of required locations. Additionally, this approach would enable the identification of optimal locations for deploying key oil skimmers.

## 5. Experimental Results

We conducted experiments utilizing a 3.6 GHz Intel Core i7-4790 CPU (quad-core) and 16 GB of memory. All methods were implemented in the C++ language.

### 5.1. Evaluation by Simulation

We executed our GA developed in Section 4.1, which uses the parameters listed in Table 1. This process took approximately 10 s. Table 3 presents the simulation results when mobilizing the vessel at a speed of 10 knots, comparing the existing assignment of South Korea with the assignment obtained from the GA. For the 16 locations, the GA-based oil skimmer assignment strategy reduces the time by 1.9% compared to the existing strategy. Moreover, when the vessel is mobilized at a speed of 5 knots, the existing assignment requires a total of 217 h. Conversely, the assignment based on the proposed method requires a total of 203 h, indicating a reduction of 0.1 h on average at each location.

**Figure 4:**
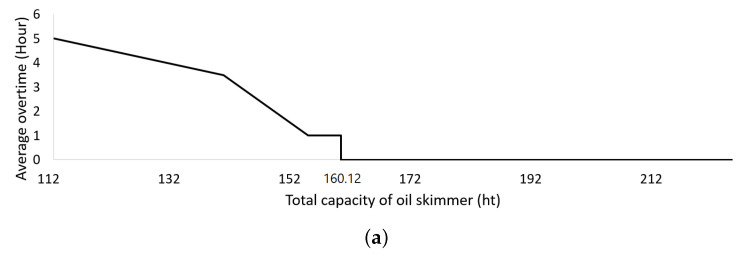

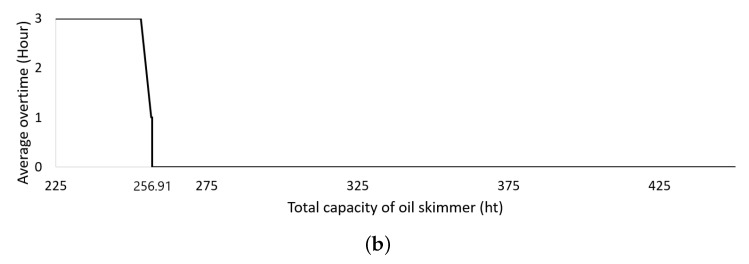
The minimum total assigned capacity derived from a binary search (the first capacities with zero overtime were 160.12 hectotonnes when mobilizing the vessel at a speed of 10 knots and 256.91 hectotonnes when mobilizing the vessel at a speed of 5 knots). (**a**) Mobilizing the vessel at a speed of 10 knots; (**b**) Mobilizing the vessel at a speed of 5 knots.

Furthermore, as depicted in Figure 4, the minimum capacity of the oil skimmers required to mobilize the vessel at a speed of 10 knots was 160.12 hectotonnes. Conducting a binary search using our GA for 450 hectotonnes, which is double the current total capacity, we obtained a reduction of 29% compared to the total capacity of the existing assignment. When the vessel was mobilized at a speed of 5 knots, it proved impossible to recover the spilled oil within the target amount of hours using the 225 hectotonne oil skimmers, the current total assigned capacity. However, with oil skimmers assigned to the 16 locations having a capacity of 256.91 hectotonnes, a 12% increase over the existing assignment, the spilled oil could be successfully recovered within 24 h, the upper limit of work time.

### 5.2. Evaluation by Surrogate Model

Two strategies were considered for the optimal oil skimmer assignment for oil spill responses, as discussed in Kim et al. [28]. The first strategy aims to minimize the anticipated work hours by reallocating the assigned oil skimmers when an oil spill accident occurs. We conducted simulations using both the GA and the surrogate-assisted GA 100 times each for this optimization strategy. The assigned amounts and resulting work hours are summarized in Table 4.

**Figure 5:**
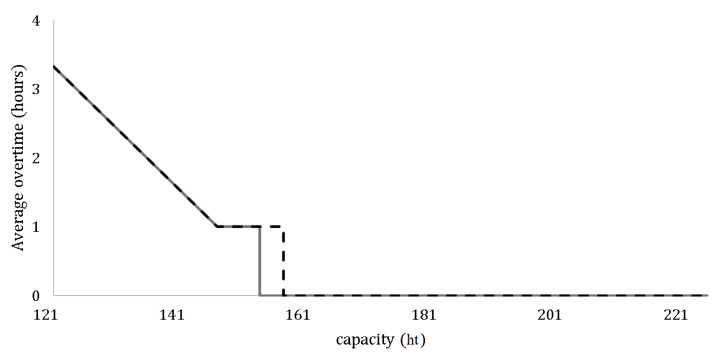
The minimized capacities of simulation-based genetic algorithm (dotted line) and surrogate-assisted one (solid line) when we mobilize the vessel at a speed of 10 knots.

The second optimization strategy aims to minimize the total assigned capacity. Using a binary search based on the current total assigned capacity of 225 hectotonnes, we determined the minimal capacity that could be derived using the surrogate-assisted GA. A comparison of the results of this minimization strategy with the simulation-based GA is illustrated in Figure 5.

### 5.3. Mobilized Location Minimization

In Figure 3, each of the 16 red and blue locations represents an assignment plan that takes into account the work start time and the vessel movement speed. The results derived from the work time minimization strategy in Section 4.1 and the mobilized location minimization strategy in Section 4.3 are listed in Table 5. Both strategies successfully recover the target amount of all 19 scenarios within the given work time without violating the 24 h constraint.

However, Table 5 reveals that, through the mobilized location minimization strategy in Section 4.3, the number of locations mobilized during the work time decreased on average in all cases, with an overall decrease of approximately 12%. Surprisingly, it was also confirmed that the overall work time decreased by approximately 7% compared to the work time minimization strategy in Section 4.1. This indicates that applying the minimization strategy to the optimization in Section 4.1 effectively reduces the problematic space to derive a good assignment.

When observing the assignment plan to which our mobilized location minimization strategy is applied, oil skimmers are mainly distributed around each of the three sea sides, confirming the combination of locations that can be key to the response of oil spill accidents in South Korea. This strategy presents a new guideline for the assignment of oil skimmers that is not available in the existing maritime accident response system in South Korea.

## 6. Concluding Remarks

For the first time, we endeavored to develop an optimal oil skimmer assignment strategy for oil spill response based on location, utilizing a simulation-based evaluation method tailored to real-world scenarios. Additionally, we introduced a genetic algorithm (GA) for the optimal oil skimmer assignment. Upon reassigning oil skimmers based on our GA results, we observed a 1.9% reduction in work time compared to the current standard assignment, assuming vessel mobilization at a speed of 10 knots. Furthermore, optimization by the GA enabled oil spill recovery within the target work hours (24 h), even with a 29% reduction in the total capacity of currently assigned oil skimmers.

Our strategy, aimed at minimizing oil-removal operation time, exhibited performance akin to the simulation-based GA, surpassing the current assignment. Moreover, the strategy to minimize the total assigned capacity demonstrated a 36% reduction compared to the current assigned capacity of 225 hectotonnes. Thus, our surrogate-assisted GA proved effective, yielding similar performance to the simulation-based GA while reducing computing time by 61%.

These findings suggest avenues for future research, including exploring scenarios more akin to real work environments and diversifying oil spill accident scenarios and equipment in experiments. Furthermore, conducting pattern analysis of multiple assignment strategies derived from optimization algorithms may yield representative assignment strategies.

While our research focused on simple scenarios, adjustments to simulate more complex and realistic scenarios significantly increased computing time due to additional constraints. In contrast, the surrogate model maintained similar speeds even with increased constraints. Thus, further exploration of the proposed surrogate model is needed. Future studies should explore adaptive sampling to reduce training data requirements, integrate traditional and deep learning models, develop real-time learning systems, implement automated hyperparameter optimization, conduct scalability studies, and investigate energy-efficient computing solutions. These efforts aim to enhance the efficiency and applicability of DNN-based surrogate models for chromosome evaluation and other computationally intensive tasks. Also, in our future work, we plan to conduct a thorough evaluation of our approach against various metaheuristic optimization techniques such as simulated annealing [29], tabu search [30], ant colony optimization [31], particle swarm optimization [32], differential evolution [33], harmony search [34], beetle antennae search [35], and egret swarm optimization [36] and perform a comprehensive comparative analysis. This will help to better understand the relative strengths and potential advantages of our method.

## Figures and Tables

**Table 1:** Parameters of the designed genetic algorithm.

Genetic Operation	Values
Selection	Roulette wheel selection [25]
Recombination	Uniform crossover [26] of rate = 0.7
Mutation	Genewise mutation of rate = 0.001
Replacement	Elitism
Population size	100
Number of generations	60,000

**Table 2:** Cross-validation results of our DNN-based surrogate model.

Measure	Values
Mean absolute error (MAE)	$2.17\times 10^{-4}$
Root mean sqaure error (RMSE)	$2.18\times 10^{-4}$
Mean work time	17.11 h

**Table 3:** Comparison of South Korea’s current assignment of oil skimmers and that obtained from our genetic algorithm.

Location (Latitude, Longitude)	Oil Spill Accident (ht)	Oil Skimmer’s Capacity (ht)		Work Time (h)	
		**Current**	**GA**	**Current**	**GA**
Incheon (37.456° N, 126.705° E)	85	28.33	34.03	9	8
Pyeongtaek (37.016° N, 126.994° E)	12	not assigned	0.5	4	4
Deasan (36.967° N, 126.421° E)	450	34	32.3	20	19
Gunsan (35.968° N, 126.737° E)	38	12.67	14.57	9	9
Mokpo (34.812° N, 126.392° E)	85	28.33	19.67	10	10
Wando (34.311° N, 126.755° E)	6	2	24.24	7	2
Yeosu (34.760° N, 127.662° E)	450	42	44.31	18	18
Jeju (33.500° N, 126.531° E)	8	2.67	1.79	8	6
Seogwipo (33.254° N, 126.560° E)	5	not assigned	0.5	9	8
Tongyeoung (34.854° N, 128.433° E)	17	5.33	2.84	5	5
Changwon (35.203° N, 128.600° E)	12	not assigned	0.5	6	7
Busan (35.210° N, 129.069° E)	25	8.33	3.71	5	6
Ulsan (35.554° N, 129.238° E)	450	57	42.82	20	20
Pohang (36.093° N, 129.305° E)	8	2.67	2.22	5	5
Donghae (37.507° N, 129.056° E)	5	1.67	0.5	9	9
Sokcho (38.176° N, 128.520° E)	0.5	not assigned	0.5	6	5
Total	1656.5	225	225	150	141

**Table 4:** Performance comparison of South Korea’s current assignment, simulation-based GA, and GA with a surrogate model using DNNs.

	Current Assignment		Simulation-Based GA		GA with DNN-Based Surrogate Model	
**Location**	**Oil Skimmer’s Capacity (ht)**	**Work Time (h)**	**Oil Skimmer’s Capacity (ht)**	**Work Time (h)**	**Oil Skimmer’s Capacity (ht)**	**Work Time (h)**
Incheon	28.33	9	17.72	8	12.62	8
Pyeongtaek	not assigned	5	0.94	4	0.11	4
Deasan	34	20	70.85	19	85.68	19
Gunsan	12.67	9	2.1	10	0.66	10
Mokpo	28.33	10	11.7	10	0.11	11
Wando	2	9	1.25	9	0.11	9
Yeosu	42	19	59.12	18	40	19
Jeju	2.67	9	1.67	9	0.11	9
Seogwipo	not assigned	9	0.91	9	0.1	9
Tongyeoung	5.33	7	3.55	6	1.81	7
Changwon	not assigned	8	2.5	8	0.77	8
Busan	8.33	6	5.22	6	0.11	6
Ulsan	57	19	45.63	20	82.59	19
Pohang	2.67	7	0.81	7	0.11	7
Donghae	1.67	9	0.92	9	0.1	9
Sokcho	not assigned	8	0.11	8	0.01	6
Total capacity	225	163	225	160	225	163
Average work time (h)	17.361		17.043		17.105	
Computing time (s)	−		5.988		2.311	

**Table 5:** Comparison of the number and work time of mobilized locations for 19 accident scenarios.

Scenario		$S_{1}$	$S_{2}$	$S_{3}$	$S_{4}$	$S_{5}$	$S_{6}$	$S_{7}$	$S_{8}$	$S_{9}$	$S_{10}$	$S_{11}$	$S_{12}$	$S_{13}$	$S_{14}$	$S_{15}$	$S_{16}$	$S_{17}$	$S_{18}$	$S_{19}$	Average
**Oil Spill (ht)**		**296**	**296**	**296**	**296**	**216**	**216**	**280**	**174**	**280**	**190**	**296**	**280**	**296**	**296**	**287**	**287**	**280**	**275**	**51**	
8:00 a.m.	Origin	22/3	21/3	20/3	20/3	14/2	15/2	19/3	13/2	16/2	18/2	20/2	19/2	19/2	19/2	19/2	20/2	12/1	18/2	15/2	17.8/2.2
10 knots	New	20/3	18/3	15/3	14/2	11/2	13/2	10/1	10/2	12/2	14/2	17/2	15/2	17/2	17/2	19/2	19/3	14/1	13/2	11/1	14.7/2.0
8:00 a.m.	Origin	21/2	17/1	17/1	17/1	19/1	15/1	18/2	12/1	18/2	13/1	17/2	13/1	17/1	17/1	21/2	22/2	17/1	17/2	14/1	16.9/1.4
5 knots	New	22/1	18/1	16/1	14/1	18/1	13/1	15/2	10/1	16/2	12/1	18/1	14/1	18/1	17/1	21/2	22/2	17/1	17/2	11/1	16.3/1.3
12:00 p.m.	Origin	12/3	19/3	19/3	19/3	15/2	18/2	20/3	11/1	17/2	15/2	18/2	16/2	18/2	18/2	20/2	21/3	12/1	17/2	12/2	16.7/2.2
10 knots	New	19/3	17/2	16/2	15/2	14/2	16/2	14/3	8/1	13/2	12/2	15/2	13/2	17/2	19/2	20/2	20/2	18/1	14/2	9/1	15.2/1.9
12:00 p.m.	Origin	22/2	22/2	20/2	21/2	23/2	19/2	17/1	13/1	19/1	12/1	12/1	11/1	17/2	16/1	24/2	24/2	16/1	15/1	15/1	17.8/1.5
5 knots	New	19/1	19/1	19/1	19/1	22/1	18/1	16/2	16/1	22/2	15/1	15/1	14/1	17/1	19/1	23/2	24/1	12/1	18/2	17/1	18.1/1.2

One can see the location of each scenario from Figure 3. In each “T/M” value, “T” is work time (h) and “M” is the number of mobilized locations.

**Figure A1:**
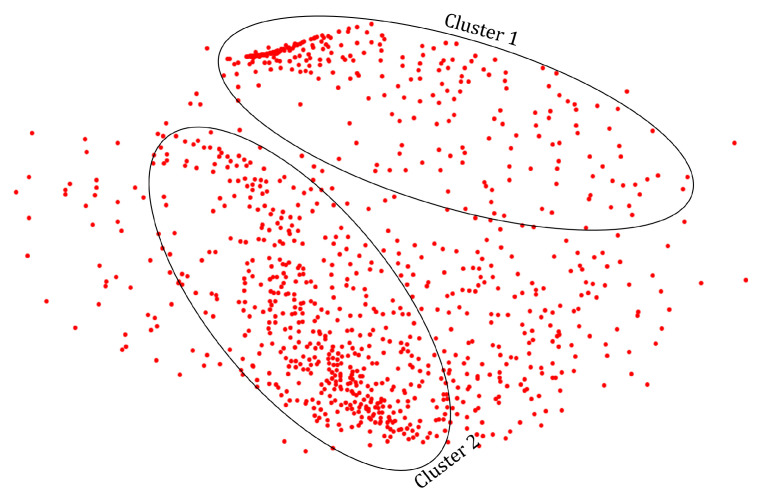
Projection results of 16-dimensional oil skimmer assignments using Sammon’s mapping [39], generated from 2000 samples.

**Figure A2:**
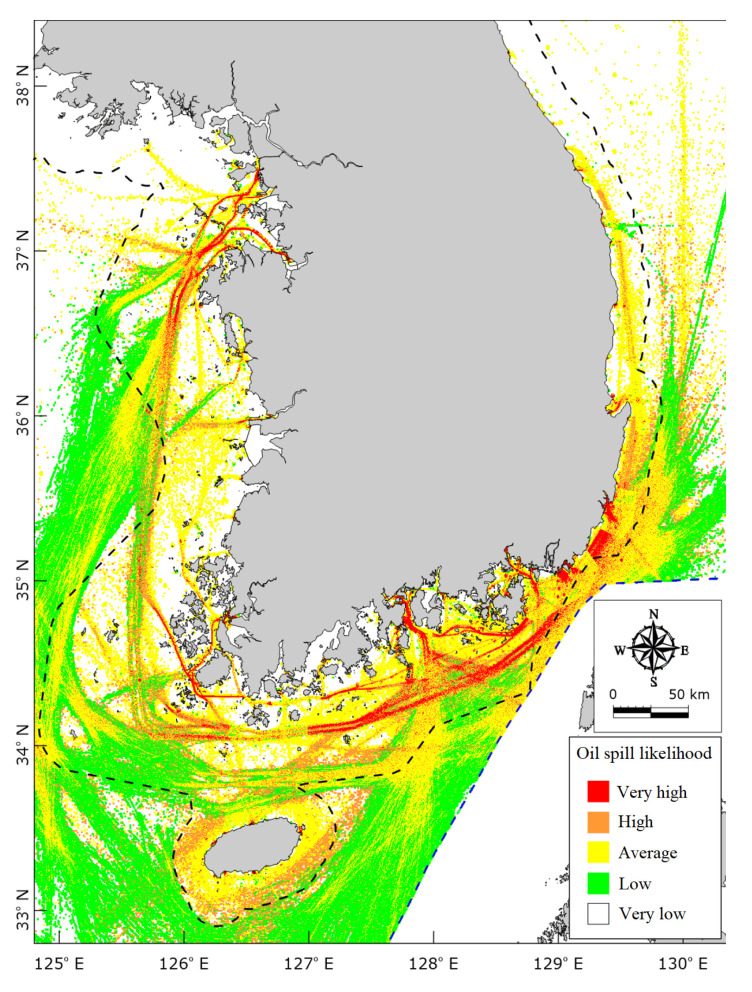
Oil spill likelihood in South Korea.

## Data Availability

The original contributions presented in the study are included in the article, and further inquiries can be directed to the corresponding author.

## Abbreviations

The following abbreviations are used in this manuscript:

GA	genetic algorithm
DNN	deep neural network
ht	hectotonne (100 tonnes)
MAE	mean absolute error
RMSE	root mean square error
AIS	Automatic Identification System
KCG	Korea Coast Guard
LHW	length × height × width
LH	length × height

## Appendix A. Clustering Analysis of Oil Skimmer Assignments

As a plan for oil spill accidents, studying and analyzing effective oil skimmer assignments is essential. However, there has been a lack of studies that diversify and analyze such assignments. In response to this need, we utilized the genetic algorithm (GA) described in Section 4.1 for optimal oil skimmer assignment. The GA produced 10,000 effective oil skimmer assignments. We then applied the *k*-means algorithm [37,38] to cluster these assignments. This clustering revealed two distinct clusters based on the assigned amounts of Yeosu, Daesan, and Ulsan, which are areas expected to experience the largest oil spills.

Additionally, we employed Sammon’s mapping [39] to project the 16-dimensional data into 2 dimensions. This allowed us to analyze the distribution of the projected data. We found that the simulation results were superior for oil skimmer assignments included in clusters compared to those not in clusters. This clustering analysis provided the basis for implementing the surrogate model described in Section 4.3.

The *k*-means algorithm is commonly used for clustering analysis. It partitions data into *k* clusters by minimizing the variance of the Euclidean distances from the center of each cluster. For our clustering analysis, we kept the vessel speed fixed at 10 knots and collected 10,000 effective assignments using our GA based on the time of accident occurrence. Through separate experiments, we found that the clusters were most distinct when *k* was set to 2.

For distribution analysis using the resulting clusters, we sampled 1000 assignments from each cluster and applied Sammon’s mapping. This technique projects high-dimensional data onto a lower dimension based on distances between instances in the original space. By applying Sammon’s mapping based on Euclidean distance, we were able to project the 16-dimensional assignment data into 2-dimensional space. Furthermore, based on density, we identified two clusters, as shown in Figure A1. Simulation results demonstrated that assignments belonging to clusters performed better than those not belonging to clusters.

In summary, we collected 10,000 effective assignments and analyzed their distributions using the *k*-means algorithm and Sammon’s mapping. By diversifying the effective assignments and analyzing their distributions, we identified two distinct patterns based on the assigned amounts in Daesan, Yeosu, and Ulsan, which are the regions with the highest potential for oil spillage. Furthermore, the assignment plans belonging to these patterns exhibited promising simulation results. Based on the results of analyzing the diversified effective assignments, we were able to design a genetic algorithm with computational efficiency through the development of surrogate models that could replace simulations [40,41,42]. The developed surrogate model consists of two separate deep neural networks (DNNs), each trained from the data of one of the two identified clusters.

## Appendix B. Scenarios of Oil Spill Accidents

GeoSystem Research Corporation (https://www.geosr.com, accessed on 27 May 2024) in South Korea derived the oil spill accident scenarios that are used in this study.

Generally, the possibility of oil spill accidents is defined by accident frequency, and, concerning oil spill accidents, the frequency is associated with the likelihood of accidents, while the scale of accidents can be assessed in terms of potential oil spillage.

In marine environments, oil spill accidents mostly occur due to accidents involving vessels in operation (such as collisions, groundings, overturning, sinking, flooding, etc.), and the extent of oil spillage during accidents is determined by the type and size of the involved vessel. Recently, there have been incidents of oil spill accidents from onshore oil storage facilities, particularly around Ulsan and Yeosu.

This study evaluates the likelihood of oil spill accidents in the South Korean maritime area using data from the past oil spill accident database (from 2000 to 2019), data from the Automatic Identification System (AIS) [43] in 2018, and oil storage facility data in 2019.

Based on a grid system established for the South Korean maritime area with a grid interval of 300 m × 300 m, accident frequencies and maximum spill volumes are derived from the past oil spill accident database (from 2000 to 2019) for each grid cell. AIS data in 2018 are utilized to determine vessel traffic density (collision risk) and oil movement density for each grid cell, while information on oil storage facilities, which comes from the database of the Korea Coast Guard (KCG) in 2019, is used to determine the oil storage capacity for each grid cell. These five indicators are integrated to assess the likelihood of oil spill accidents, as shown in Figure A2. The assessment area encompasses all the jurisdictions under the KCG responsible for oil spill response.

The thickness (or concentration) of spilled oil and its impact range in the event of maritime oil spill accidents are influenced by accident conditions (such as the location, type, and volume of the spill) and environmental conditions (such as tides, currents, winds, etc.). Therefore, when formulating accident scenarios, realistic combinations of these conditions are necessary to consider the feasibility and methods of containment. In this study, to derive the potential impact range of oil spills for the South Korean maritime area and to incorporate it into the assessment of environmental risks posed by oil spills, accident conditions are constructed based on past oil spill cases, vessel traffic density (collision risk), oil movement density, and oil storage facility capacity. Recent five-year marine and weather conditions are considered to select the timing of oil spill accidents and construct oil spill accident scenarios:

- Past oil spill accident records (frequency and spill volume): analyzing data from the past oil spill accident database (from 2000 to 2019) based on a grid system (300 m × 300 m) established for the South Korea maritime area to derive accident frequencies and maximum spill volumes for each grid cell;
- Vessel traffic density (collision risk): to understand the likelihood of vessel accidents closely related to oil spill accidents, vessel traffic density (frequency) for each grid cell is derived from AIS data for 2018 at hourly intervals, and cumulative annual vessel traffic for each grid cell is calculated. Additionally, analysis of collision risk is performed, defining collision risk as the sum of collision risks for different encounter situations (head-on collision, crossing collision, rear-end/overtaking collision), with higher collision risks indicating a higher probability of oil spill accidents;
- Oil movement density: estimation of oil movement density for each grid cell is conducted based on AIS data at hourly intervals for 2018;
- Oil storage facilities (storage capacity): analyzing data on oil storage facilities from the KCG’s database in 2019 for the South Korean maritime area, based on the grid system of 300 m × 300 m, allows us to derive the oil storage capacity for each grid cell. High-capacity oil storage facilities (with storage capacities of 1000 hectotonnes or more) are primarily distributed around major ports such as Incheon North, Pyeongtaek, Daesan, Yeosu, Busan, and Ulsan;
- Estimation of spill volume: since most maritime oil spill accidents are caused by vessels in operation and spill volumes are influenced by the size of the vessel, the maximum dimensions of vessels at accident locations are identified using AIS information. Vessel sizes are then determined, and spill volumes are calculated by distinguishing between tanker and non-tanker vessels.
